# G protein-coupled receptor GPR160 is associated with apoptosis and cell cycle arrest of prostate cancer cells

**DOI:** 10.18632/oncotarget.7313

**Published:** 2016-02-10

**Authors:** Caihong Zhou, Xinchuan Dai, Yi Chen, Yanyan Shen, Saifei Lei, Ting Xiao, Tamas Bartfai, Jian Ding, Ming-Wei Wang

**Affiliations:** ^1^ The National Center for Drug Screening and The CAS Key Laboratory of Receptor Research, Shanghai Institute of Materia Medica, Chinese Academy of Sciences (CAS), Shanghai 201203, China; ^2^ The State Key Laboratory of Drug Research, Shanghai Institute of Materia Medica, Chinese Academy of Sciences (CAS), Shanghai 201203, China; ^3^ Department of Chemical Physiology, The Scripps Research Institute, La Jolla, CA 92037, USA; ^4^ School of Pharmacy, Fudan University, Shanghai 201203, China

**Keywords:** GPR160, prostate cancer, orphan G protein-coupled receptor, cell cycle arrest, apoptosis

## Abstract

G protein-coupled receptors (GPCRs) represent the largest membrane protein family implicated in the therapeutic intervention of a variety of diseases including cancer. Exploration of biological actions of orphan GPCRs may lead to the identification of new targets for drug discovery. This study investigates potential roles of GPR160, an orphan GPCR, in the pathogenesis of prostate cancer. The transcription levels of GPR160 in the prostate cancer tissue samples and cell lines, such as PC-3, LNCaP, DU145 and 22Rv1 cells, were significantly higher than that seen in normal prostate tissue and cells. Knockdown of GPR160 by lentivirus-mediated short hairpin RNA constructs targeting human *GPR160* gene (ShGPR160) resulted in prostate cancer cell apoptosis and growth arrest both *in vitro* and in athymic mice. Differential gene expression patterns in PC-3 cells infected with ShGPR160 or scramble lentivirus showed that 815 genes were activated and 1193 repressed. Functional annotation of differentially expressed genes (DEGs) revealed that microtubule cytoskeleton, cytokine activity, cell cycle phase and mitosis are the most evident functions enriched by the repressed genes, while regulation of programmed cell death, apoptosis and chemotaxis are enriched significantly by the activated genes. Treatment of cells with *GPR160*-targeting shRNA lentiviruses or duplex siRNA oligos increased the transcription of *IL6* and *CASP1* gene significantly. Our data suggest that the expression level of endogenous GPR160 is associated with the pathogenesis of prostate cancer.

## INTRODUCTION

G protein-coupled receptors (GPCRs) are cell-surface molecules that transduce extracellular signals into intracellular effector pathways through the activation of heterotrimeric G proteins [1]. Owing to their special structural features, signal transduction pathways and extensive physiological functions, GPCRs rank the highest success rate among all drug target categories in pharmaceutical development [2]. About 40% clinically approved drugs target GPCRs [3]. There are more than 800 members of GPCRs, but only a small number of them are targeted by current drugs [4]. A tremendous amount of efforts have been made so far aiming at exploiting therapeutic applications of the remaining family members, including more than 140 orphan GPCRs whose endogenous ligands have yet to be unmasked [5].

Since the first identification of *MAS* gene, which encodes a functional GPCR, as an oncogene [6], an increasing body of evidence links GPCR expression and activation to human primary and metastatic tumors [7, 8]. GPCRs, G proteins and their downstream signaling affect different facets of human malignancies, including cancer initiation and progression, cell invasion and metastasis, angiogenesis, as well as the establishment and maintenance of a permissive microenvironment [8]. Widespread mutations of G proteins and GPCRs were also found in common cancer cells, such as activating mutations of *GNAS* (encoding Gα_s_) in 28% of growth hormone-secreting pituitary tumors and 5% of thyroid adenomas, that of *GNAQ* or *GNA11* (encoding Gαq) in 66% or 6% of melanomas, as well as that of thyroid stimulating hormone receptor (TSHR) gene in thyroid cancer, just to name a few [9]. Signal transduction of GPCRs and crosstalk of downstream signals, including second messengers, Ras and Rho GTPases, mitogen-activated protein kinases (MAP kinases), phosphoinositide-3 kinases (PI3Ks), and numerous associated cytosolic and nuclear targets, contribute to cell growth, survival, differentiation and migration. Malignant cells are capable of hijacking such normal functions to advance their growth. Thus, understanding the roles that GPCRs play in human malignancies would certainly help the discovery of novel therapeutic agents.

Orphan GPCR is a rich source of potential drug targets. Tremendous efforts have been made to de-orphanize them or to study their signaling mechanisms and potential functions [5]. GPR160 is an orphan class A GPCR previously annotated as GPCR1 or GPCR150. The human GPR160 protein is of 338-amino acid long and encoded by 7 exons located at 3q26.2-q27 [10]. Orthologues of GPR160 have been identified in the Rhesus monkey, dog, cow, rat, mouse, chicken, zebrafish, and frog. The rodent GPR160 has 336 amino acids and shares about 65% homology with that of the human. While the transcription level of *GPR160* gene in different tissues varies dramatically from the reproductive system (especially in the testes) showing the most abundance, its mRNA in humans is mainly distributed in the small intestine, duodenum, colon, bone marrow, kidney, bladder and prostate [11, 12]. Up-regulation of *GPR160* transcription was found in many human cancer cell lines or tissue samples. In 2005, Schlomm *et al.* reported differential *GPR160* expression between cancerous and normal prostate duct cells [13]. An aberrantly higher expression of GPR160 in CD4^+^CD56^+^ hematodermic neoplasm was noted [14]. Amplification of *GPR160* at 3q26.2-q26.32 was also detected in two nasopharyngeal carcinoma cell lines [15], an observation consistent with that seen in metastatic melanoma as opposed to benign samples [16]. It is known that malignant cells are dependent on constitutive or overexpression of driver genes [17], which may be regulated by microRNAs (miRNAs) [18]. The expression of *GPR160* in lymphoblastoid cells was negatively controlled by miR-125b, but its effect on the receptor function has yet to be identified [19].

Prostate cancer is currently the most commonly diagnosed non-dermatologic malignancy among males and the second leading cause of death in North America and Europe [20]. Though androgen ablation has temporary and limited beneficial effects on the control of androgen-dependent tumors, there is an unmet medical need for novel therapeutic modalities for advanced and metastatic prostate cancer, such as monoclonal antibodies, T cell-mediated immunotherapy or novel chemical compounds with better target selectivity. An increasing number of GPCRs have been implicated in neoplastic transformation of the prostate. Overexpression of prostate-specific G protein-coupled receptor (PSGR) [21, 22] and bradykinin 1 receptor [23] was observed in prostate cancer cells. GPRC6A, a class C GPCR, was proposed recently as a target for the control of prostate growth and cancer progression [24, 25]. In addition, activation of cysteine (C)-X-C receptor 4 (CXCR4) together with its ligand CXCL12 promotes ligand-independent activation of androgen receptor, and the latter is responsible for prostate cancer metastasis [26, 27]. Therefore, identification of new biomarkers or new targets is a crucial step to improve diagnosis and treatment of this deadly disease. We compared the expression profile of a series of orphan GPCRs between normal prostate tissues and prostate cancer samples and found one of them, GPR160, showed different expression patterns. The aims of this study are to analyze the expression profile and cellular function of GPR160 in the context of target validation for prostate cancer.

## RESULTS

### Overexpression of GPR160 mRNA in prostate cancer tissue and cells

**Figure 1:**
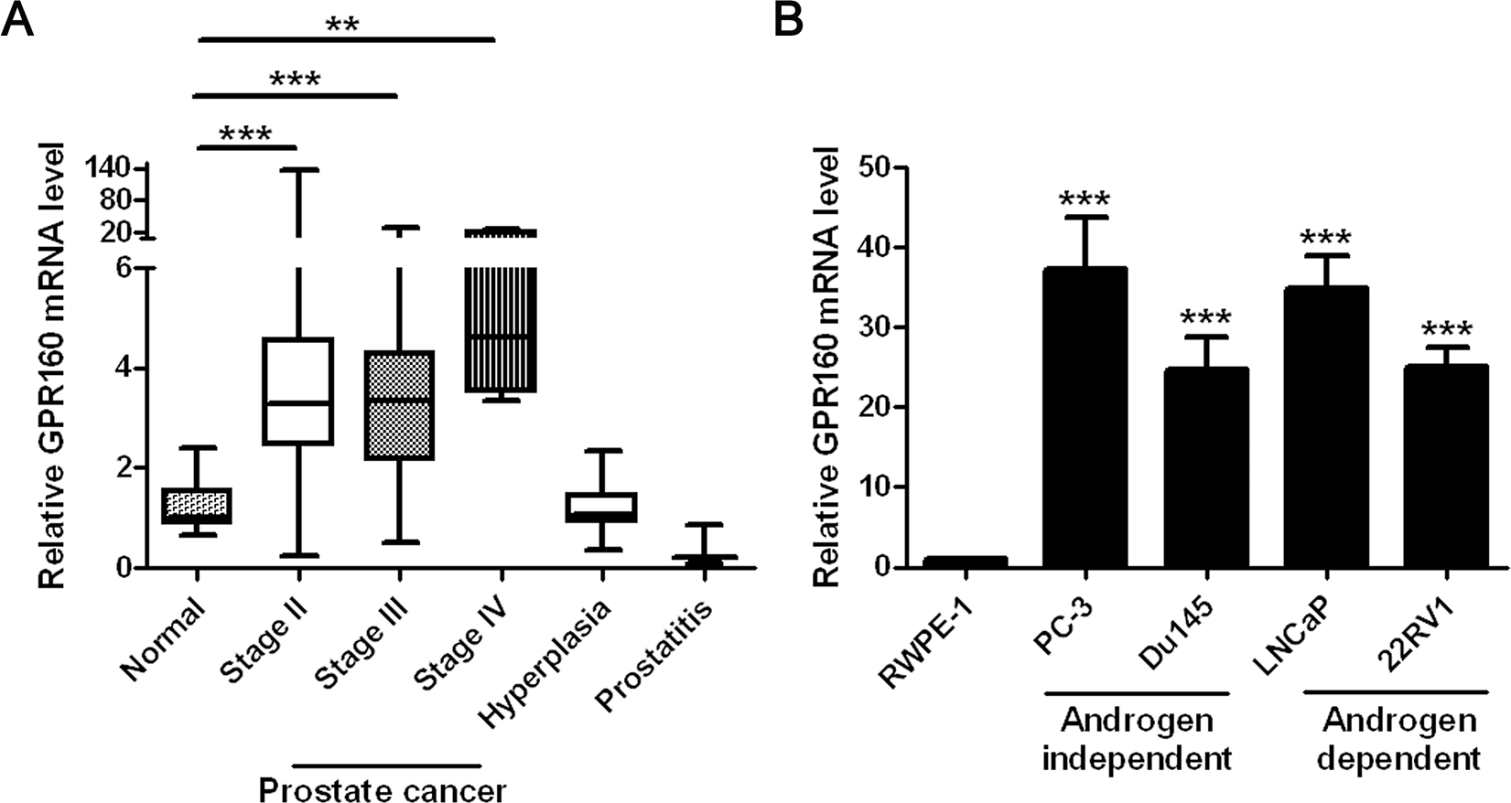
Expression of GPR160 in human prostate cancer tissue samples and cell lines (**A**) GPR160 gene expression in prostate cancer samples assessed by cDNA array and qPCR analysis of combined histological groups [box (interquartile range) and whiskers (min-max) plot with median (central bar)]. (**B**) Relative GPR160 mRNA expression in prostate cancer cell lines. For each sample, qPCR was conducted for GPR160 normalized against reference TATA-binding protein (TBP) gene (*n =* 3). **P < 0.01 and ***P < 0.001 compared with normal prostate tissue samples or RWPE-1 cells.

In order to screen orphan GPCRs that are associated with prostate cancer, we queried the Gene Expression Omnibus (GEO) [28] and patent databases [29] for clues of candidate genes. GPR160 was found to be up-regulated in primary and metastatic prostate cancer samples (Supplementary Figure S1, GEO accession GDS2546) [30]. To confirm this, a batch of prostate tissue cDNA arrays, derived from tissue samples of normal, prostate lesion or prostate cancer patients, was used in conjunction with quantitative RT-PCR (qRT-PCR). It was found that the *GPR160* mRNA levels in all stages of prostate cancer samples were significantly higher than that of normal, but comparable among different cancer stages (Figure 1A). The average *GPR160* transcription level in prostate hyperplasia tissues was similar to that of normal but in prostatitis tissue samples it was lower than normal, though no statistical significance was noted due to a small sample size (*n* = 3). *GPR160* mRNA could hardly be detected in RWPE-1 cells, an established cell line derived from normal prostate epithelial cells transfected with a single copy of the human papilloma virus 18 (HPV-18) [31]. A much higher level of *GPR160* expression was observed in human prostate cancer cells such as PC-3, DU145, LNCaP and 22Rv1 (Figure 1B). PC-3 and DU145 cells are androgen-independent whereas the growth of LNCaP and 22Rv1 is dependent upon androgen, suggesting that GPR160 is not essential to androgen-mediated cellular events.

### Suppression of PC-3 and LNCaP cell growth by GPR160 knockdown

**Figure 2:**
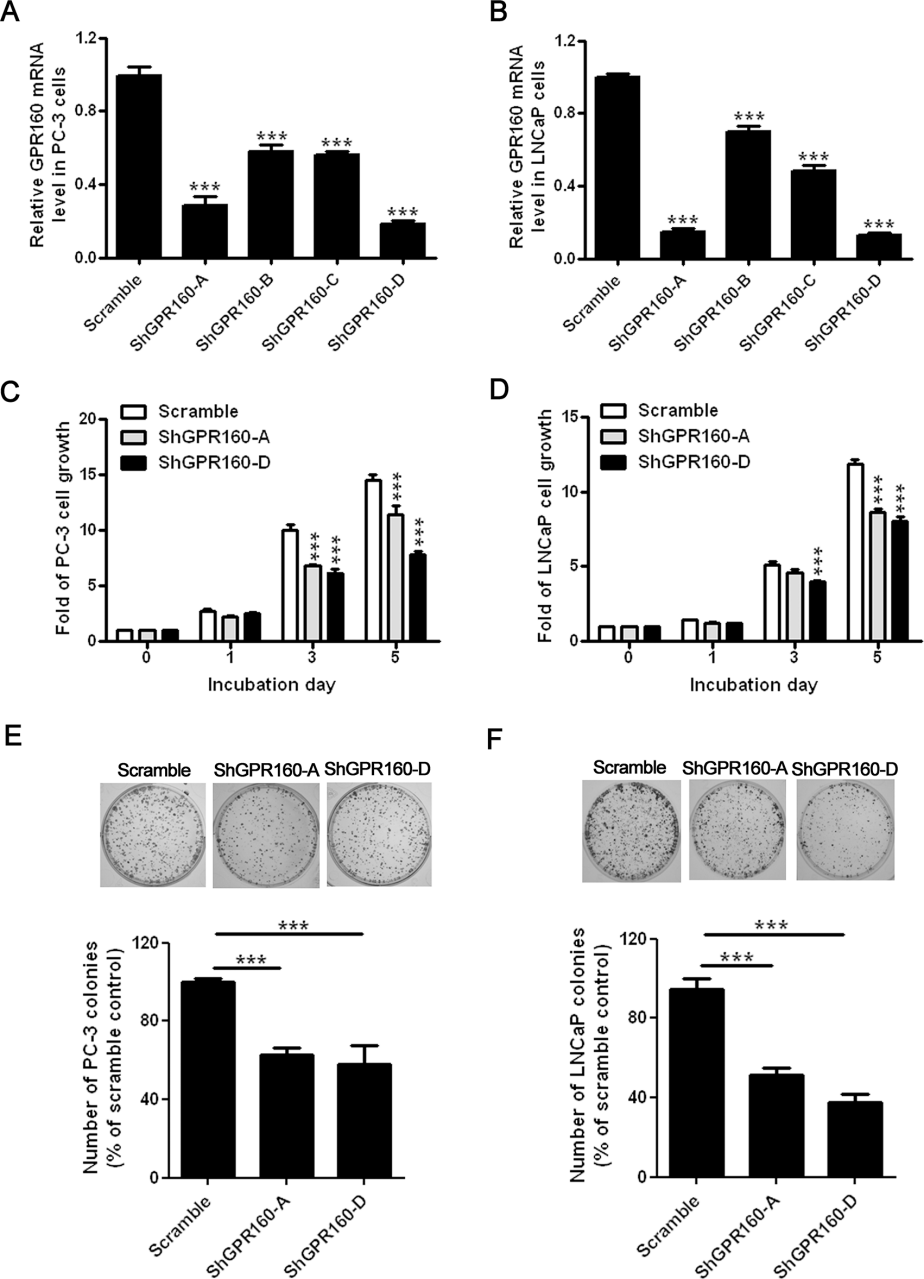
Suppression of prostate cancer cell growth by knockdown of endogenous GPR160 *in vitro* *GPR160* gene was silenced with shRNA lentiviruses, namely, ShGPR160-A, B, C and D. The expression level of GPR160 mRNA was detected with qRT-PCR in PC-3 (**A**) and LNCaP (**B**) cells. (**C** and **D**) Two days after lentivirus infection, cells were collected and reseeded in 96-well plates at a density of 2500 cells per well for PC-3 and 5000 cells per well for LNCaP cells. Cell growth was monitored with Cell Counting Kit 8. (**E** and **F**) Colony formation of ShGPR160-infected cells. The top panels are representative pictures for PC-3 (**E**) and LNCaP (**F**) cell clones and the bars represent relative colony count of three independent experiments with triplicate wells. Data are presented as means ± s.e.m. of at least 3 independent experiments. ***P < 0.001 compared with scramble virus infected cells.

Gene knockdown is now a widely used technique to study gene functions. To study the effect of GPR160 knockdown on the growth of prostate cancer cells, we produced a lentivirus system containing either scramble shRNA (scramble) or four different shRNAs targeting human *GPR160* (ShGPR160-A, B, C and D). LNCaP and PC-3 cells were selected for their relatively higher GPR160 mRNA levels and covering both androgen dependent and independent growth properties. The efficiency of lentivirus infection was determined by flow cytometry analysis of GFP expression three days after infection. More than 80% infected cells were GFP-positive. The silencing efficiency of ShGPR160 was examined with qRT-PCR. A reduction of GPR160 mRNA level by 71.4% in ShGPR160-A-treated or by 81.3% in ShGPR160-D-treated PC-3 cells was achieved; similarly, decreases of 84.8% in ShGPR160-A-treated and 84.9% in ShGPR160-D-treated LNCaP cells were detected, compared to scramble treatment (Figure 2A and 2B).

ShGPR160-A and ShGPR160-D were then employed to study the effect of GPR160 knockdown on cell growth. When cell proliferation was assessed with Cell Counting Kit-8 through consecutive culture for 5 days, a clear-cut reduction in metabolically active cells was observed in ShGPR160-treated compared to scramble-treated cells (Figure 2C and 2D). In both cell lines the most pronounced effects were seen on day 5. A colony formation assay was subsequently conducted to confirm the above observation. As shown in Figure 2E, colony formation of GPR160 knockdown cells was decreased to 61 ± 10% for ShGPR160-A and to 58 ± 23% for ShGPR160-D, respectively, in PC-3 cells compared with the control. In LNCaP cells, infection with ShGPR160-A and ShGPR160-D led to a decrease of colony count to 53 ± 11% and 37 ± 13%, respectively (Figure 2F). These data demonstrate that shRNAs targeting GPR160 suppressed the growth of both PC-3 and LNCaP cells.

### Inhibition of tumor formation by GPR160 knockdown *in vivo*

**Figure 3:**
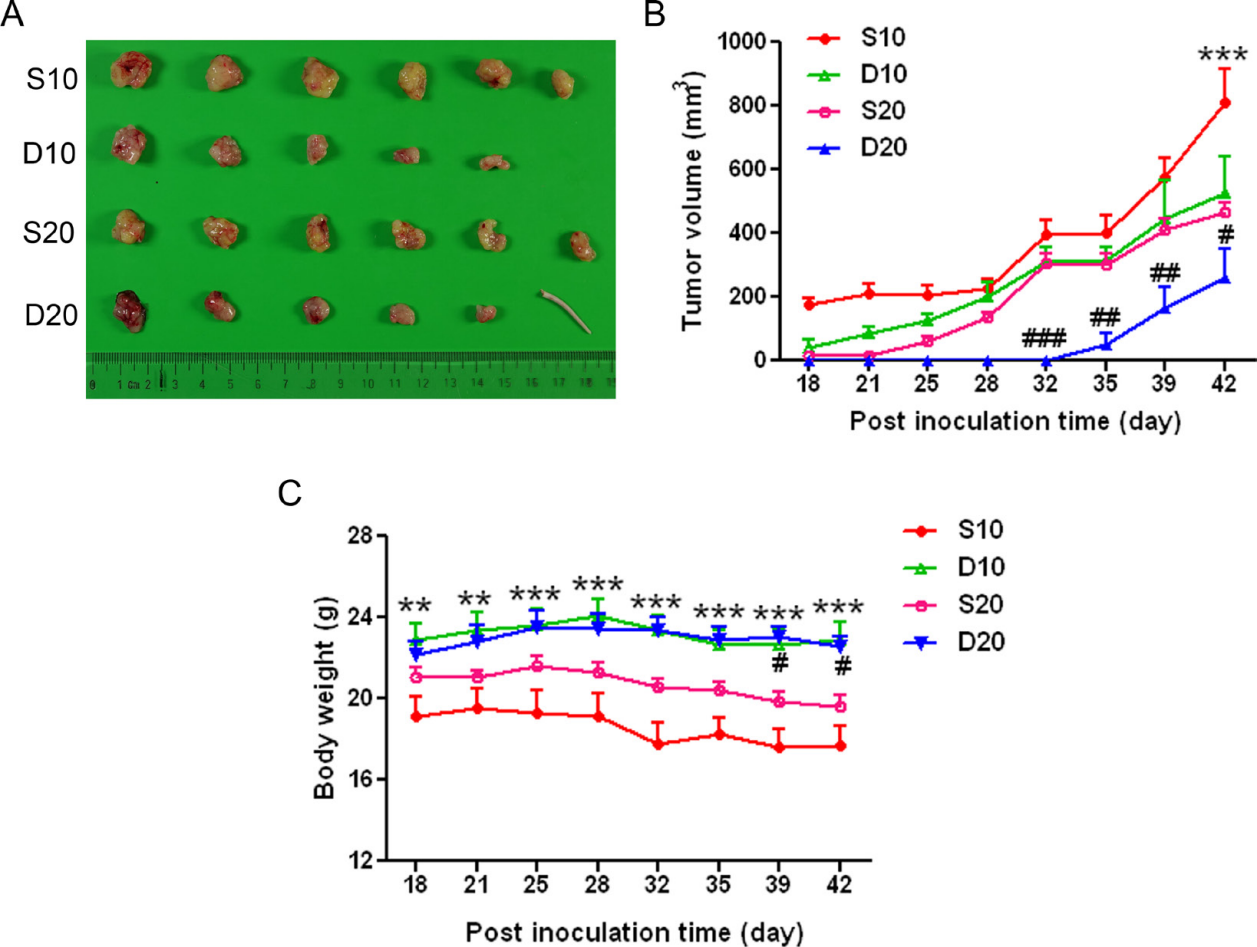
Transduction of GPR160-targeting shRNA lentiviruses attenuated the growth of PC-3 xenografts in athymic nude mice PC-3 cells were infected with scramble or ShGPR160-D lentiviruses and then subcutaneously (*s.c.*) injected into the right flank of nude mice at 4 × 10^7^ cells/mouse (five or six mice per group). (**A**) is gross tumor appearances 42 days after cell inoculation and (**B**) depicts tumor volume. (**C**) Body weight of treated animals. Data are presented as means ± s.e.m. ^#^*P* < 0.05, **^,##^*P* < 0.01 and ***^,###^*P* < 0.001 compared with scramble control groups.

To assess the effect of GPR160 expression on cell proliferation *in vivo*, PC-3 cells infected with scramble or ShGPR160-D lentiviruses were subcutaneously inoculated into nude mice. Almost all mice developed tumors within 42 days after inoculation, however, the silencing of GPR160 impaired tumor growth. As shown in Figure 3A, when cells infected with ShGPR160 at an multiplicity of infection (MOI) of 20 (D20), the growth of tumors in nude mice was much slower than that of the cells treated with ShGPR160 at an MOI of 10 (D10) and scramble controls (S10 and S20). The average tumor volume of D20 group was 260 ± 223 mm^3^ on day 42, significantly smaller than that of S20 group (467 ± 71 mm^3^, *P* < 0.05), while that of D10 was 525 ± 256 mm^3^, also significantly smaller than that of the S10 group (810 ± 260 mm^3^, *P* < 0.001). The body weight of the mice was less affected in D20 and D10 groups compared to S20 and S10 controls (Figure 3B).

### Induction of apoptosis and cell cycle arrest by GPR160 knockdown

**Figure 4:**
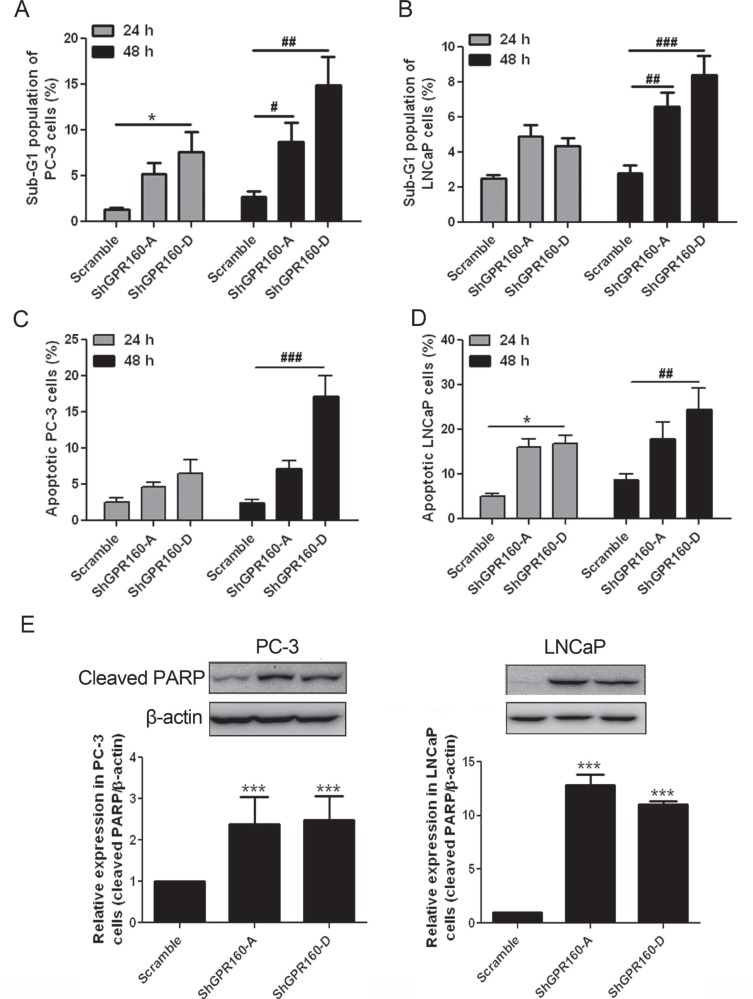
Induction of apoptosis by GPR160 knockdown (**A** and **B**) Sub-G_1_ peak analysis of PC-3 and LNCaP cells after ShGPR60 infection. Scramble and ShGPR160 lentivirus-infected cells were seeded and incubated for 24 h or 48 h before collection and fixation with ethanol. After PI staining, sub-G1 fraction was analyzed by flow cytometry. (**C** and **D**) Detection of APC-Annexin V labeled cells in ShGPR160-infected PC-3 and LNCaP cells. (**E**) Knockdown of GPR160 induced the cleavage of PARP in PC-3 and LNCaP cells. Data are presented as means ± s.e.m. of at least 3 independent experiments. *^,#^*P* < 0.05, ^##^*P* < 0.01 and ***^,###^*P* < 0.001 compared with scramble control.

Apoptosis of ShGPR160-infected cells was first determined with sub-G_1_ peak analysis after propidium iodide (PI) staining. The population of Sub-G_1_ cells increased significantly in ShGPR160-infected than those in scramble-treated cells (Figure 4A and 4B).

Confirmation of ShGPR160-induced apoptosis was carried out with Annexin V staining and cleaved poly (ADP-ribose) polymerase (PARP) analysis. In allophycocyanin (APC)-conjugated Annexin V staining assay, ShGPR160 treatment resulted in a significant increase of Annexin V positive cell population compared with the scramble control both in PC-3 and LNCaP cells (Figures 4C and 4D). The expression of 89-kDa cleaved PARP protein is a marker of apoptotic cells [32]. In PC-3 cells, cleaved PARP expression was significantly enhanced by ShGPR160-A (2.8-fold, *P* < 0.05) and ShGPR160-D (2.5-fold, *P* < 0.05) compared with the control, while in LNCaP cells, the increase was more pronounced exhibiting 12.8-fold (*P* < 0.001) and 9.0-fold (*P* < 0.001) elevation for ShGPR160-A and ShGPR160-D, relative to the control, respectively (Figure 4E).

Since apoptosis induced by GPR160 knockdown was evident in cells with high levels of endogenous GPR160 expression, such as LNCaP and PC-3, we expanded our investigation to 22RV1 cells which displayed a relatively low level of endogenous GPR160 expression. Following continuous incubation upon lentivirus infection with ShGPR160-A or ShGPR160-D, morphological alteration associated with a markedly increased number of resting 22RV1 cells was observed (Supplementary Figure S2), indicative of cell cycle arrest.

### Identification of genes potentially targeted by GPR160

**Figure 5:**
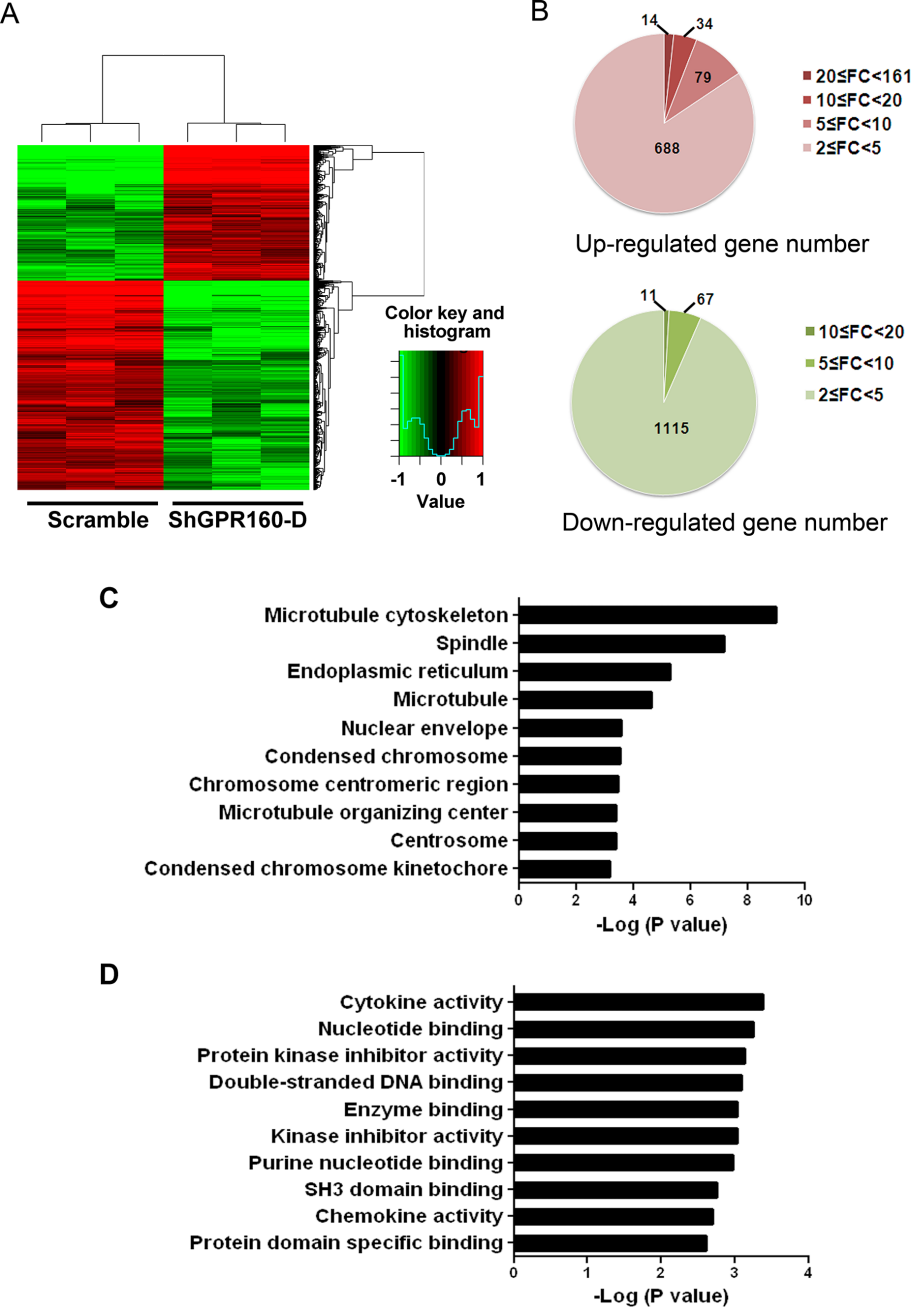
Microarray data for differentially expressed genes (DEGs) between scramble and ShGPR160 infected PC-3 cells (**A**) Heat map showing DEGs in ShGPR160 infected cells was generated using normalized log 2-transformed values as shown in pseudo color scale with red indicating activated transcript level and green representing repressed expression of a specific gene. (**B**) Up- and down-regulated gene numbers annotated. FC is the abbreviation of fold change. Gene Ontology analysis of cellular components (**C**) and molecular functions (**D**) were presented for genes modified upon ShGPR160 treatment in PC-3 cells.

To elucidate genes and pathways potentially targeted by GPR160 in prostate cancer cells, differentially expressed genes (DEGs) between scramble- and ShGPR160-treated PC-3 cells were analyzed with Affymetrix GeneChip Human Genome U133 Plus 2.0 Array. This microarray contains 47000 probes representing 38500 Entrez genes. A total of 2008 genes were found to display differential expression between ShGPR160 and scramble treatment with a selection criterion of fold change ≥ 2 (*P* < 0.05). Hierarchical clustering of the dysregulated genes exhibited a similar expression pattern within biological replicates and distinct differences between the two groups (Figure 5A). There were 815 up-regulated genes potentially indirectly related to GPR160 and 1193 down-regulated genes possibly directly linked with GPR160 (Figure 5B). The top 20 genes with the most significant changes of transcription are listed in Supplementary Tables S1 and S2. Changes in mRNA expression of listed genes upon ShGPR160 treatment with *P* values less than 0.001 in Supplementary Table S1 was confirmed by qRT-PCR. The results are consistent with that obtained from the GeneChip analysis (Supplementary Figure S3).

### Putative functions of DEGs in ShGPR160-treated cells

To gain insights into the functional changes between scramble- and ShGPR160- infected PC-3 cells, we employed the Database for Annotation, Visualization and Integrated Discovery (DAVID) online tool to analyze the pathway enrichment. As shown in Table 1, the most significantly enriched pathway of DEGs (fold change ≥ 2, *P* < 0.05) was hsa04060 (cytokine-cytokine receptor interaction, *P* < 0.05). Three of the top 10 pathways, including hsa04310 (Wnt signaling pathway), hsa05210 (colorectal cancer) and hsa04110 (cell cycle), were associated with cancer. They were all repressed upon GPR160 knockdown. Four pathways, including hsa04060 (cytokine-cytokine receptor), hsa04630 (Jak-STAT signaling pathway), hsa04623 (cytosolic DNA-sensing pathway) and hsa04621 (NOD-like receptor signaling pathway), were activated.

**Table 1:** The enriched KEGG pathway of DEGs

Term	Pathways name	Count	Size	*P* value	Fold enrichment	Status
**hsa04060**	Cytokine-cytokine receptor interaction	39	262	5.80E-10	3.04	A
**hsa04114**	Oocyte meiosis	21	110	2.18E-05	2.92	I
**hsa04630**	Jak-STAT signaling pathway	20	155	1.63E-04	2.64	A
**hsa04310**	Wnt signaling pathway	23	151	2.73E-04	2.33	I
**hsa05210**	Colorectal cancer	16	84	2.88E-04	2.91	I
**hsa04110**	Cell cycle	20	125	4.13E-04	2.44	I
**hsa04623**	Cytosolic DNA-sensing pathway	10	55	1.20E-03	3.71	A
**hsa04621**	NOD-like receptor signaling pathway	10	62	2.83E-03	3.29	A
**hsa04810**	Regulation of actin cytoskeleton	26	215	3.13E-03	1.85	I
**hsa04720**	Long-term potentiation	12	68	4.13E-03	2.69	I

Notes: Count: The number of DEGs. Size: The total number of genes in the pathway. Status: A, activated; I, inhibited.

Abbreviations: KEGG, Kyoto Encyclopedia of Genes and Genomes; DEGs: differentially expressed genes.

Gene ontology (GO) analysis is a common approach for functional analysis of large-scale genomic or transcriptomic data. The represented GO categories in biological process were analyzed with a threshold of *P* value less than 0.05 and gene count larger than 2. Major cellular components associated with ShGPR160 treatment in PC-3 cells was illustrated in Figure 5C. Microtubule cytoskeleton is the most involved organelle. Among the top 10 GO molecular function categories, cytokine activity (GO:0005125) ranked the first followed by nucleotide binding (GO:0000166), protein kinase inhibitor activity (GO:0004860) and double-stranded DNA binding (GO:0003690) (Figure 5D).

Functional annotation of the repressed and activated genes was then interrogated, respectively, using DAVID. The results revealed that the top biological process enriched by genes that were repressed with ShGPR160 treatment is cell cycle phase (GO:0022403) followed by mitosis (GO:0007067; Table 2). The crucial processes activated include regulation of programmed cell death (GO:0043067), apoptosis (GO:0006915), chemotaxis (GO:0006935), negative regulation of programmed cell death (GO:0043069) and regulation of leukocyte activation and proliferation (GO:0002696 and GO:0070663) (Table 2). The top 20 genes of the 81 DEGs repressed in cell cycle phase are listed in Table 3. Most of these genes are encoding proteins associated with the G2 phase (*CDC25C*, *NEK2*, *CENPF* and *CENPE*) and M phase (*CIT*, *ASPM*, *SGOL2*, *KIF23*, *UBE21*, *TNKS* and *CCNB2*), indicating the major impact of GPR160 on the G2/M phase of cell cycle. The top 20 up-regulated genes of the 71 DEGs associated with positive regulation of programmed cell death are listed in Table 4. Caspases are the key players in apoptosis [33]. As exhibited in Table 4, the most differentially expressed caspase gene upon ShGPR160 treatment is caspase 1 (*CASP1*), whose expression was increased by 7.64-fold (*P =* 0.002).

**Table 2:** Major biological processes modified by ShGPR160 infection in PC-3 cells

Term	Biological process	Count	Size	*P* value	Fold enrichment	Status
**GO:0022403**	Cell cycle phase	81	414	1.18E-20	3.17	I
**GO:0007067**	Mitosis	55	220	4.52E-19	4.05	I
**GO:0043067**	Regulation of programmed cell death	71	812	2.10E-08	2.02	A
**GO:0006915**	Apoptosis	54	602	6.54E-07	2.07	A
**GO:0006935**	Chemotaxis	24	160	3.97E-07	3.46	A
**GO:0043069**	Negative regulation of programmed cell death	36	359	6.23E-06	2.31	A
**GO:0002696**	Positive regulation of leukocyte activation	17	106	1.23E-05	3.70	A
**GO:0070663**	Regulation of leukocyte proliferation	15	84	1.35E-05	4.12	A
**GO:0009952**	Anterior/posterior pattern formation	24	140	1.45E-05	2.78	I
**GO:0051338**	Regulation of transferase activity	44	372	5.03E-05	1.92	I

Notes: Count: The number of differentially expressed genes. Size: The total number of genes in the biological process. Status: A, activated; I, inhibited.

**Table 3:** DEGs in the cell cycle process in ShGPR160 treated PC-3 cells

Symbol	Gene name	*P* value	Fold change
**LFNG**	LFNG O-fucosylpeptide 3-beta-N-acetylglucosaminyltransferase	0.0076	−10.68
**FOXN3**	Forkhead box N3	0.0018	−5.58
**NCAPD2**	Non-SMC condensin I complex, subunit D2	0.0002	−4.72
**CENPF**	Centromere protein F, 350/400 ka (mitosin)	0.0029	−4.11
**CDC25C**	Cell division cycle 25 homolog C (*S. pombe*)	0.0174	−3.91
**NEK2**	NIMA (never in mitosis gene a)-related kinase 2	0.0018	−3.82
**CIT**	Citron (rho-interacting, serine/threonine kinase 21)	0.0088	−3.60
**CCNB1**	Cyclin B1	0.0011	−3.59
**CDKN2B**	Cyclin-dependent kinase inhibitor 2B (p15, inhibits CDK4)	0.0005	−3.57
**GSPT1**	G_1_ to S phase transition 1	0.0124	−3.56
**CENPE**	Centromere protein E, 312 kDa	0.0002	−3.49
**MPHOSPH9**	M-phase phosphoprotein 9	0.0130	−3.44
**ASPM**	Asp (abnormal spindle) homolog, microcephaly associated (Drosophila)	0.0059	−3.41
**DLGAP5**	Discs, large (Drosophila) homolog-associated protein 5	0.0000	−3.33
**PAFAH1B1**	Platelet-activating factor acetylhydrolase, isoform Ib, subunit 1 (45 kDa)	0.0093	−3.26
**SGOL2**	Shugoshin-like 2 (*S. pombe*)	0.0085	−3.25
**KIF23**	Kinesin family member 23	0.0121	−3.19
**UBE2I**	Ubiquitin–conjugating enzyme E2I (UBC9 homolog, yeast)	0.0002	−3.18
**TNKS**	Tankyrase, TRF1–interacting ankyrin-related ADP-ribose polymerase	0.0001	−3.17
**CCNB2**	Cyclin B2	0.0003	−3.13

Abbreviations: DEGs: Differentially expressed genes.

**Table 4:** DEGs in the biological process of regulation of programmed cell death in ShGPR160-treated PC-3 cells

Symbol	Gene name	*P* value	Fold change
**SERPINB2**	Serpin peptidase inhibitor, clade B (ovalbumin), member 2	0.0001	59.50
**IL6**	Interleukin 6 (interferon, β2)	0.0001	21.75
**CARD16**	Caspase recruitment domain family, member 16	0.0033	13.83
**TNFAIP3**	Tumor necrosis factor, α-induced protein 3	0.0001	12.77
**BCL2A1**	BCL2-related protein A1	0.0001	9.33
**CD38**	CD38 molecule	0.0426	9.32
**DUSP1**	Dual specificity phosphatase 1	0.0009	9.02
**ADRB2**	Adrenergic, β-2-, receptor, surface	0.0003	8.57
**IDO1**	Indoleamine 2,3-dioxygenase 1	0.0040	7.79
**CASP1**	Caspase 1, apoptosis-related cysteine peptidase (interleukin 1, beta, convertase)	0.0020	7.64
**F3**	Coagulation factor III (thromboplastin, tissue factor)	0.0001	7.13
**FOSL1**	FOS-like antigen 1	0.0024	6.35
**IFI16**	Interferon, γ-inducible protein 16	0.0057	6.23
**IL12A**	Interleukin 12A (natural killer cell stimulatory factor 1, cytotoxic lymphocyte maturation factor 1, p35)	0.0003	6.03
**TNFSF10**	Tumor necrosis factor (ligand) superfamily, member 10	0.0033	5.88
**PLCG2**	Phospholipase C, γ2 (phosphatidylinositol-specific)	0.0008	5.82
**CDKN1A**	Cyclin-dependent kinase inhibitor 1A (p21, Cip1)	0.0004	5.29
**CLCF1**	Cardiotrophin-like cytokine factor 1	0.0019	4.99
**PMAIP1**	Phorbol-12-myristate-13-acetate-induced protein 1	0.0007	4.80
**SOD2**	Superoxide dismutase 2, mitochondrial	0.0019	4.71

Abbreviations: DEGs: Differentially expressed genes.

**Figure 6:**
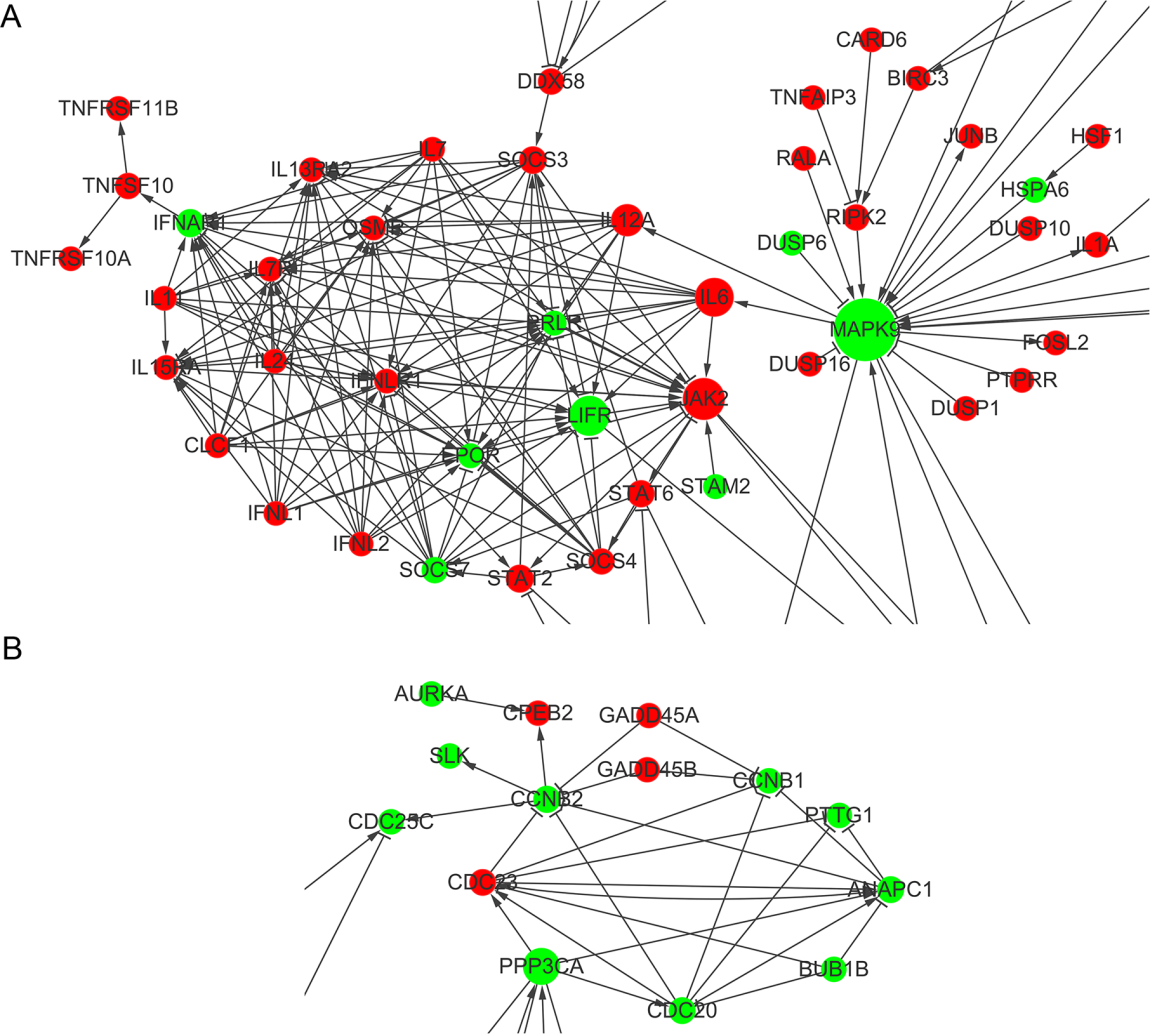
Gene interaction network analysis of differentially expressed genes between scramble and ShGPR160-D treated PC-3 cells (**A**) Interactive cytokine and cytokine receptor genes. (**B**) Interactive genes in the cell cycle biological process. Network maps were generated using Cytoscape with main genes marked as nodes and edges representing relation types between the nodes.

Network analysis revealed that many of genes altered by ShGPR160-D treatment are connected to kinases, such as *PRKCA*, *PIK3R3*, *MAPK9*, *PLCB1* and *PRKACA*, whose expression were all down-regulated. A group of cytokine and cytokine receptors were found to be tightly connected, with *MAPK9* linking to *IL12A* and *IL6*, both increased *JAK2* expression (Figure 6A). In terms of cell cycle related biological process, *CCNB1*, *CCNB2* and *CDC25C* were found to be connected in the network (Figure 6B).

### Confirmation of DEG expression and caspase activation

**Figure 7:**
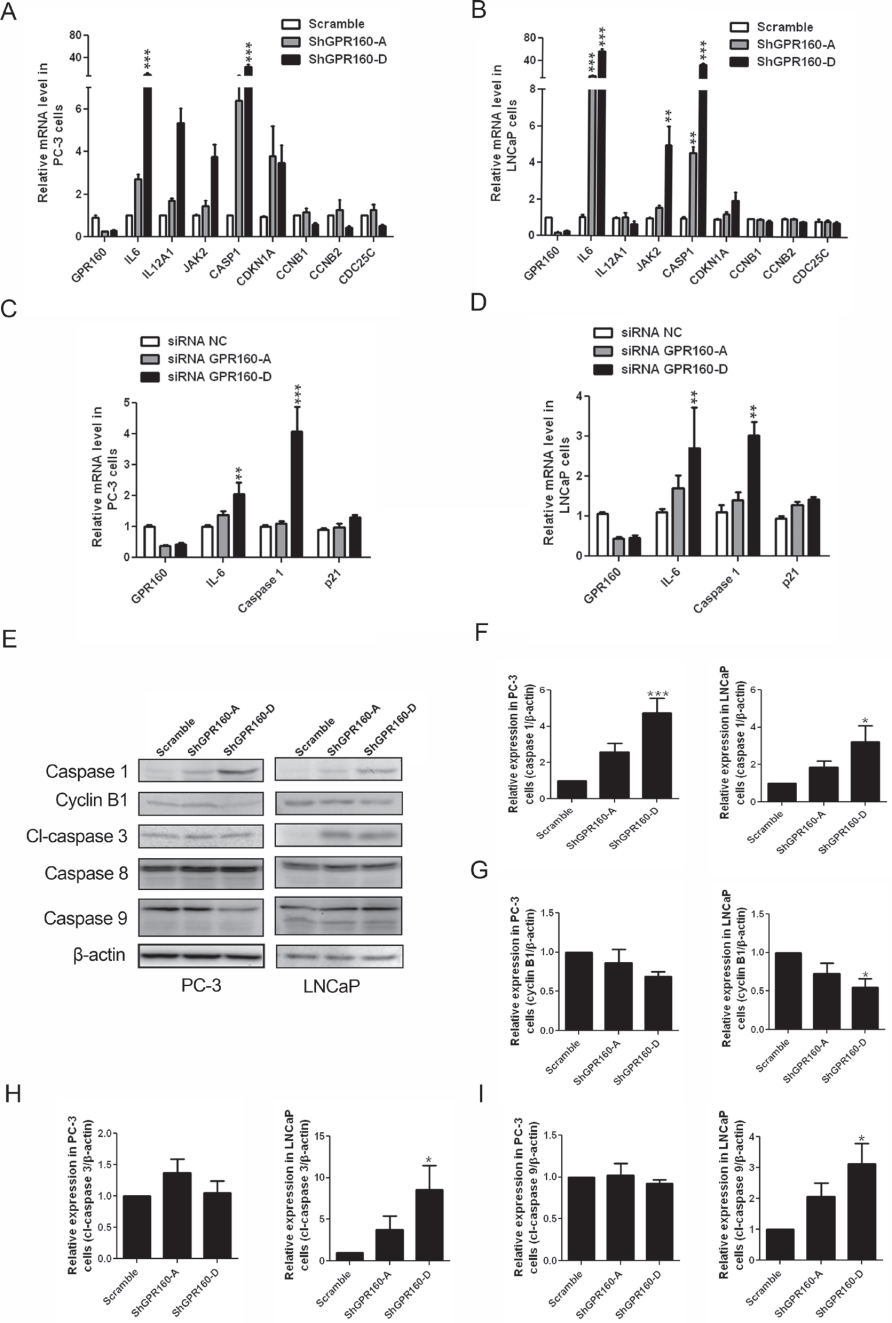
Confirmation of differentially expressed genes between scramble and ShGPR160-D treated PC-3 and LNCaP cells and caspase activation (**A** and **B**) qRT-PCR confirmation of genes involved in the regulation of apoptosis and cell cycle process in ShGPR160-treated prostate cancer cells. (**C** and **D**) Effects of GPR160-targeting oligo siRNAs on mRNA levels of DEGs. (**E**–**I**) Effects of ShGPR160 treatment on caspase activation and cyclin B1 expression. Cells were collected 5 days after lentivirus transduction and then subjected to Western blot analysis. Bars represent means ± s.e.m. of at least 3 independent experiments. *P < 0.05, **P < 0.01 and ***P < 0.001 compared with scramble virus-infected cells. cl, cleaved.

Expression of DEGs in the context of cell cycle and programmed cell death was further examined with qRT-PCR. Significantly elevated expression of *CASP1* was confirmed upon ShGPR160 treatment, *i.e.*, increased by 6 ± 3 times for ShGPR160-A and 23 ± 17 times for ShGPR160-D in PC-3 cells and by 5 ± 1 times for ShGPR160-A and 35 ± 5 times for ShGPR160-D in LNCaP cells, respectively. The expression of *IL6* was increased by 2.7 ± 0.8 times for ShGPR160-A and 10 ± 8 times for ShGPR160-D in PC-3 cells and by 14 ± 5 times for ShGPR160-A and 57 ± 9 times for ShGPR160-D in LNCaP cells, respectively. Increased expression of *CDKN1A* and *JAK2* was also detected accompanied by decreased expression of *CCNB1, CCNB2* and *CDC25C* in these cells (Figure 7A and 7B). Increased expression of *IL6* and *CASP1* is known to be involved in the cellular response to viral infection [34]. In order to determine if changes in *IL6* and *CASP1* expression was associated with the lentivirus-mediated gene knockdown system, we synthesized double-stand siRNA oligos with the same core sequences as ShGPR160-A and ShGPR160-D. After transient transfection of GPR160 siRNAs in PC-3 cells, increases of *IL6* and *CASP1* mRNA levels were 1.4 ± 0.3 and 1.1 ± 0.2 folds for siRNA GPR160-A, 2.0 ± 0.9 and 4.0 ± 0.8 folds for siRNA GPR160-B in comparison with non-specific control (siRNA NC), respectively (Figure 7C). In LNCaP cells, the expression of *IL6* and *CASP1* was increased by 1.7 ± 0.7 and 1.4 ± 0.4 folds for ShGPR160-A, 2.7 ± 2.3 and 3.0 ± 0.7 folds for ShGPR160-D, respectively (Figure 7D), implying that the increased expression of *IL6* and *CASP1* was induced by *GPR160* silencing specifically.

The production of caspase 1 protein was analyzed by Western blot using an anti-caspase 1 antibody. As depicted in Figure 7E and 7F, ShGPR160 treatment increased the caspase 1 protein levels by 2.6 ± 0.8 times for ShGPR160-A and 4.7 ± 2.0 times for ShGPR160-D in PC-3 cells and by 2.0 ± 0.6 times for ShGPR160-A and 2.8 ± 1.5 times for ShGPR160-D in LNCaP cells, respectively. The proteins encoded by *CCNB1* and *CCNB2* are cyclins B1 and B2, both can bind phosphorylated cell division cycle protein 2 homolog (cdc2) to regulate G2/M transition of cell cycle. The decreased expression of cyclin B1 was also confirmed with anti-cyclin B1 antibody using Western blot analysis (Figure 7G).

To ascertain that caspase 1 is the major caspase involved in apoptosis induced by GPR160 knockdown, we examined the effects of selective caspase 1 inhibitor, Belnacasan, also known as VX-765, on cell viability upon ShGPR160 treatment. At 1 μM, Belnacasan rescued PC-3 cells from apoptosis by increasing the cell viability from 83 ± 10% to 105 ± 23% for ShGPR160-A and from 81 ± 11% to 97 ± 21% for ShGPR160-D, respectively (Supplementary Figure S4). The activation of caspases 1, 3/7, 8 and 9 was also investigated in both PC-3 and LNCaP cells. When evaluated with a luminescent caspase 3/7 activity assay, we found that the activity of caspase 3/7 was increased significantly upon ShGPR160 treatment in LNCaP cells (by 3.4 ± 1.2 folds for ShGPR160-A and 6.8 ± 3.3 folds for ShGPR160-D) in comparison with the scramble control. Only a moderate increase was seen in PC-3 cells (Supplementary Figure S5). Expression and activation of caspase 8 did not change whereas elevated production of cleaved caspase 3 (Figure 7H) and cleaved caspase 9 (Figure 7I) proteins was noted in LNCaP cells but not in PC-3 cells compared to the scramble control. This suggests that, besides caspase 1, other caspases also play a role in apoptosis induced by *GPR160* silencing and the action varies under different cellular microenvironment.

## DISCUSSION

The main objective of this study was to determine if GPR160 plays a role in the pathogenesis of prostate cancer. Investigation of transcription profiles of *GPR160* demonstrated a marked increase of GPR160 mRNA levels in all stages of prostate cancer samples and cancer cell lines, but not in prostate hyperplasia tissues. This is consistent with a previous report showing up-regulation of GPR160 in cancerous prostate duct cells, CD4^+^CD56^+^ hematodermic neoplasm, metastatic melanoma and nasopharyngeal carcinoma cells [13–16]. The level of GPR160 was comparable in prostate samples collected from stages II to IV cancer patients. Since this was seen in both androgen-dependent and androgen-independent cancer cell lines, we postulate that the effect of GPR160 does not require the participation of androgen receptors. Our data also suggest that GPR160 may represent a growing number of GPCRs that are up-regulated in primary and metastatic cancers to promote tumor formation [8, 35]. The less than normal level of GPR160 presence in prostatitis tissues remains to be confirmed with additional tissue samples. This line of research may help us reveal the role of GPR160 in infection and inflammation.

In the absence of cognate ligands, we could neither modulate the activity of GPR160 nor study its function effectively. However, our lentivirus-mediated shRNA system efficiently suppressed *GPR160* transcription and arrested the growth of PC-3 and LNCaP cells (Figure 2). When inoculated into nude mice, PC-3 cells carrying shRNAs targeting *GPR160* were unable to form tumors at a regular pace (Figure 3). Two MOIs were employed in this study in order to offset variability of infection with lentivirus. Both regimens (MOI10 and MOI20) developed tumors significantly smaller than that of scramble controls, suggesting an inhibitory action of *GPR160*-targeting shRNAs on tumorgenesis in PC-3 cells. The efficiency of *GPR160* silencing was confirmed by quantitative RT-PCR though the effectiveness of GPR160 knockdown could not be evaluated with Western blot analysis owing to lack of appropriate antibodies, despite we tried many times using antibodies either from commercial sources or raised in-house.

Cell apoptosis was evaluated with sub-G_1_ peak, Annexin V-staining and immunoblot analysis of cleaved PARP. In order to explore the mechanism by which GPR160 knockdown suppressed the growth of prostate cancer cells both *in vivo* and *in vitro*, Affymetrix GeneChip was applied to scramble- and ShGPR160-treated PC-3 cells to study changes of gene expression profile upon *GPR160* silencing. Our GO analysis points to two activated biological processes, *i.e*., regulation of programmed cell death and apoptosis. Caspase 1 was found to be up-regulated by 7.6-fold in GeneChip analysis that was confirmed by qRT-PCR and specific antibodies in both PC-3 and LNCaP cells (Figure 7). ShGPR160-induced apoptosis in PC-3 cells was rescued by Belnacasan, a caspase 1 selective inhibitor (Supplementary Figure S4), indicating a key role of caspase 1 in this process. Caspases are proteolytic enzymes largely known for their functions in controlling cell death and inflammation. Caspases 2, 3, 7, 8, 9 and 10 are of apoptotic nature, whereas caspases 1, 4, 5, 11 and 12 are involved in inflammation [36]. Caspase 1 was reported to have tumor suppressor properties and is frequently down-regulated in human cancers, prostate cancer in particular [37, 38]. Over-expression of caspase 1 enhances the sensitivity of androgen-independent prostate cancer cells to radiation-induced death [39]. Though the proenzyme forms of caspases 1, 3 and 9 was found to be constitutively expressed in PC-3, DU-145 and LNCaP cell lines, the expression level of caspase 1 was marginal in less tumorigenic DU-145 and LNCaP cells [38]. In this study, we found that GPR160 knockdown induced apoptosis in both PC-3 and LNCaP cells, but marked activation of caspases 3 and 9 were observed only in LNCaP cells. The underlying mechanism of such a difference remains elusive.

The top molecular function enriched with GO analysis indicates that GPR160 has cytokine-like activity, which is consistent with the Network analysis showing a most tightly connected cytokine and cytokine receptor group including *IL6*, *IL12A* and *JAK2*. Transcription of *IL6* increased significantly after *GPR160* silencing. IL-6 exerts dual actions: while it elicits acute phase response and stimulates proliferation or differentiation in many cell types including B cells, thymocytes, T cells and hepatocytes, it also inhibits cell growth and induces apoptosis in some myeloma cell lines. In LNCaP cells, IL-6 induces the activation of signal transducer and activator of transcription 3 (STAT3) thus leading to enhanced neuroendocrine differentiation [40]. PC-3 cells express high levels of IL-6, which also stimulates the cell via an autocrine mechanism [41]. Pro- or anti-proliferative effects of IL-6 on prostate cancer cells may thus depend on the cellular microenvironment. Expression of both *CASP1* and *IL6* genes could be induced not only by viral infection directly but also by GPR160 siRNAs in a virus-free transient transfection system. It appears that GPR160 protein may be constitutively expressed and exerts inhibitory action on the expression of caspase 1 and IL-6. Involvement of GPR160 in cytokine expression and cytokine receptor interaction is a novel observation and warrants further investigation.

Both KEGG pathway and biological process enrichment analyses demonstrate that cell cycle was repressed by ShGPR160 treatment while mitosis ranked the second in the inhibited biological processes. About one third of the top 20 DEGs in the cell cycle process are associated with mitosis. Among them, most are connected with G_2_/M cell phase such as *CCNB1*, *CCNB2* and *CDC25C*. Network analysis also indicates a connection among *CCNB1*, *CCNB2* and *CDC25C*. However, cyclin B1 protein did not change significantly in ShGPR160-treated PC-3 cells after a long-time culture. Considering the morphological alteration upon *GPR160* silencing observed in 22Rv1 cells, there may exist other mechanisms to regulate cell cycle besides cyclins B1 and B2.

Taken together, our observations highlight GPR160 as a candidate target for the treatment of prostate cancer. Knockdown of GPR160 in prostate cancer cells increased the expression of caspase 1 and IL-6, induced cell cycle arrest and apoptosis, though the underlying molecular mechanism remains to be identified. Analysis of DEGs between scramble- and ShGPR160-treated prostate cancer cells suggests that GPR160 is also associated with cytokine and cytokine receptor interaction. In the xenograft experiment, we found that the development of tumor in shGPR160-D treated nude mice was markedly delayed in comparison to scramble controls. The involvement of GPR160 in carcinogenicity of the prostate and its therapeutic implication would certainly open a new avenue for diagnostic and pharmaceutical exploration.

## MATERIALS AND METHODS

### Reagents

Dulbecco's modified Eagle's medium (DMEM), RPMI 1640 medium and K-SF medium were procured from Life Technologies (Carlsbad, CA, USA). Fetal bovine serum (FBS) was bought from Hyclone (Logan, UT, USA). Anti-β-actin, anti-caspase 1, anti-cleaved caspase 3 and anti-cleaved PARP antibodies were the products of Cell Signaling Technologies (Danvers, MA, USA). Anti-caspase 8 and anti-caspase 9 antibodies were procured from Beyotime Biotechnology Incorporation (Jiangsu, China). All restriction enzymes, DNA polymerase and DNA ligation kits were purchased from TaKaRa Biotechnology Co., Ltd. (Dalian, China). DNA purification after electrophoresis was done with TIANgel Mini Purification Kit (Tiangen Biotech Co., Ltd., Beijing, China). PCR products were purified with AxyPrep PCR Clean-up Kit (Axyegen, Union City, CA, USA). All chemical reagents were obtained from Sigma Chemical Co. (St. Louis, MO, USA). The siRNA oligos were synthesized by GenePharma (Shanghai, China). The transfection reagent Megatran 1.0 was bought from OriGene Technologies, Inc. (Rockville, MD, USA).

### Vectors and cell lines

Expression vectors containing shRNAs targeting human GPR160 in the pGFP-C-shLenti backbone were procured from OriGene. Prostate cancer cell lines PC-3, DU145, LNCaP and 22Rv1, as well as normal prostate cell line RWPE-1, were purchased from American Tissue Culture Collection (ATCC, Manassas, VA, USA). PC-3, DU145, LNCaP, and 22Rv1 cells were maintained in RPMI1640 medium containing 10% FBS, 2 mM L-glutamine, 100 μg/ml streptomycin and 100 unit/ml penicillin at 37°C and 5% CO_2_. RWPE-1 cells were grown in K-SF medium containing 50 μg/ml bovine pituitary extract and 5 ng/ml epidermal growth factor (Thermo Fisher Scientific, Rockford, IL, USA). Human embryonic kidney epithelial cell line HEK293T was maintained in DMEM supplemented with 10% FBS.

### Quantitative RT-PCR

Human prostate tissue cDNA arrays were obtained from OriGene and quantitative PCR analysis for human GPR160 was performed in each well with Taqman primer/probes according to the manufacturer's instruction. Quantitative PCR was performed on a ViiA 7 Real-Time PCR System (Applied Biosystems, Foster City, CA, USA). Cycle threshold differences of the human GPR160 were quantified relative to TATA-box binding protein (TBP) that was used as an internal control.

To examine gene expression in prostate cancer cell lines, total RNA from human prostate cancer and RWPE-1 cells was isolated with TRIzol reagent (Thermo). Two μg of DNase-treated total RNA was reverse-transcribed into cDNA with oligo dT primers and High Capacity cDNA Reverse Transcription Kit (Applied Biosystems). Reactions were carried out at 25°C for 11 min, 37°C for 2 h followed by 85°C for 5 min and 4°C for 5 min. Primers of the selected genes were listed in Supplementary Table S1. Relative quantification of gene transcription was performed with SYBR Premix DimerEraser (TaKaRa) using the comparative CT method with *ACTB* as the control. Melting dissociation was performed to evaluate the purity of the PCR product.

### Lentivirus preparation, infection and flow cytometry analysis

HEK293T cells were transfected with corresponding pGFP-C-shLenti vectors, the packaging plasmid psPAX2 and the envelope plasmid pMD2.G (Addgene, Cambridge, MA, USA) using Megatran 1.0 reagents. The viral particles were harvested 72 h thereafter and the cells (1 × 10^5^) were infected at different dilutions of viruses together with 6 μg/ml polybrene (Sigma). The expression of EGFP or GPR160-EGFP after lentivirus infection was detected by fluorescence-activated cell sorting (FACS) with an Accuri C6 cytometer (BD Biosciences, Franklin Lakes, NJ, USA).

### Cell proliferation assay

Cell proliferation assay was performed as previously described with minor modifications [42]. Briefly, cells were collected 3 days after lentivirus infection, seeded in 96-well microtiter plates at a density of 5000 cells per well for PC-3 and 2500 cells per well for LNCaP, respectively. Four hours before the end of incubation, 10 μL per well of Cell Counting Kit-8 reagent (Dojindo Laboratories, Kumamoto, Japan) was added to cells and absorbance at 450 nm measured with a FlexStation^III^ (Molecular Devices, Sunnyvale, OR, USA).

### Colony formation assay

To examine the effect of GPR160 knockdown on cell growth, PC-3 and LNCaP cells were infected with either control reagent (non-infection control, NC), lentivirus containing the scramble shRNA (scramble) or GPR160 shRNA (ShGPR160). Cells were seeded in 6-well plates at a density of 1000 cells per well for PC-3 and 4000 cells per well for LNCaP 3 days after infection. Following incubation at 37°C for 10–14 days, the colonies were fixed and stained in a dye solution containing 0.1% crystal violet (Sigma) and 20% methanol. The number of colonies per well was then counted.

### Detection of caspase 3/7 activity

The scramble or ShGPR160-infected cells were seeded at a density of 500/well in triplicate in a 384-well plate. After overnight incubation, the medium was replaced with RPMI1640 supplemented with 0.2% FBS and incubated for another 48 h. Caspase activity was subsequently measured with a Caspase-Glo 3/7 Assay System (Promega, Fitchburg, WI, USA) according to the manufacturer's protocol. Briefly, an equal volume of caspase substrate was added to the cells followed by incubation at room temperature for 1 h. The luminescence was measured using an EnVision 2103 Multilabel Reader (PerkinElmer, Waltham, MA, USA). The luminescence of untreated control cells was set as the standard.

### Western blot analysis

Cells were washed with cold phosphate-buffered saline (PBS) and lysed in ice-cold buffer. The protein concentration was determined with the Bradford protein assay (Bio-Rad Laboratories, Hercules, CA, USA). Protein extracts were loaded onto 8% or 12% SDS-PAGE and transferred to a PVDF membrane (Millipore, Billerica, MA, USA). The membrane was blocked in 5% fat-free milk and incubated with primary antibodies at 4°C overnight. After washing with PBS T (PBS with 0.05% Tween-20), the membranes were incubated with horseradish peroxidase-conjugated secondary antibodies. The protein signals were visualized with an enhanced chemiluminescence immunoblotting detection kit (LI-COR Biosciences, Nebraska, NE, USA). Actin was used as an equal loading control.

### Flow cytometry

The percent of cells undergoing apoptosis and the different phases of the cell cycle were determined by flow cytometry as previously described [43]. Cells grown in regular growth medium for 24 h or 48 h were collected, fixed in 70% cold ethanol overnight and stained with PBS containing 50 μg/ml PI and 100 μg/ml RNase A (Tiangen) for 30 min at room temperature. The DNA content of the labeled cells was measured using the Accuri C6 flow cytometry system. Apoptotic cells were defined as those in the sub-G0/G1 peak. For the detection of Annexin V positive cells, harvested cells were washed once in cold PBS and resuspended in 100 μL 1 × Annexin-binding buffer. After that, 5 μl of APC-conjugated Annexin V solution was added to each 100 μL of cell suspension. After incubation at room temperature for 15 min, cells were diluted in 400 μL 1 × Annexin-binding buffer and analyzed by Accuri C6 Flow Cytometer (BD Biosciences). Data were analyzed with FlowJo flow cytometry analysis software (Tree Star, Inc., Ashland, OR, USA).

### Animal studies

Male athymic BALB/c nude mice, 4–6 weeks old, were housed and maintained under specific-pathogen free conditions with a 12 h light/dark cycle at 25 ± 1°C and received food and water *ad libitum*. All experiments were performed according to the institutional ethical guidelines on animal care and approved by the Institute Animal Care and Use Committee at Shanghai Institute of Materia Medica (approval number: 2015–04-DJ-17). PC-3 cells were subcutaneously (*s.c.*) injected into the right flank of nude mice at 4 × 10^7^ cells/mouse (five or six mice per group). Tumor diameters were measured two times per week and tumor volumes (V) calculated using 1/2 × length × width^2^.

### Microarray analysis

Scramble and ShGPR160 lentivirus-infected PC-3 cells were collected and their gene expression profiles were displayed by Shanghai Biotechnology Corporation (China) using the Affymetrix GeneChip Human Genome U133 Plus 2.0 Array. Each group had 3 independent replicates and for each sample 1 × 10^7^ cells were collected and analyzed separately. Genespring was employed to determine feature intensities and ratios (including background subtraction and normalization). A *P* value (*P* < 0.05) and a fold-change threshold FC ≥ 2.0 were chosen to identify statistically significant transcript alterations. The DAVID online tool was used to analyze the enrichment in the Gene Onthology (GO) and KEGG Pathway among the statistically significant genes between scramble and ShGPR160 lentirus-infected PC-3 cells. GO enrichment analysis for differentially expressed genes was performed with Gene Ontology Enrichment Analysis Software Toolkit GOEAST (Institute of Genetics and Developmental Biology, Chinese Academy of Sciences, Beijing, China). Heat maps were presented using Cluster 3.0 and the TreeView software (Barcelona, Spain). Network analysis was performed to construct and visualize molecular interaction networks using the MySQL Workbench with sources of the interaction database from KEGG [44]. Network maps were generated using Cytoscape with main genes marked as nodes and edges representing relation types between the nodes [45].

### Statistical analysis

Analysis of Variance (ANOVA) was performed using GraphPad Prism software (GraphPad, San Diego, CA, USA). *P* values below 0.05 were considered significant (*P < 0.05; **P < 0.01; ***P < 0.001). Bars and error bars in the histograms represent mean values ± s.e.m. of at least three independent experiments.

## SUPPLEMENTARY MATERIALS FIGURES AND TABLES


