# Supplementary material for: G protein-coupled receptor GPR160 is associated with apoptosis and cell cycle arrest of prostate cancer cells
Source: Oncotarget. 2016 Feb 10;7(11):12823–39. doi: 10.18632/oncotarget.7313 (PMC4914324; doi:10.18632/oncotarget.7313)
Supplement: Supplementary file 1 [file oncotarget-07-12823-s001.pdf]

G protein-coupled receptor GPR160 is associated with apoptosis and cell cycle arrest of prostate cancer cells

Supplementary Materials

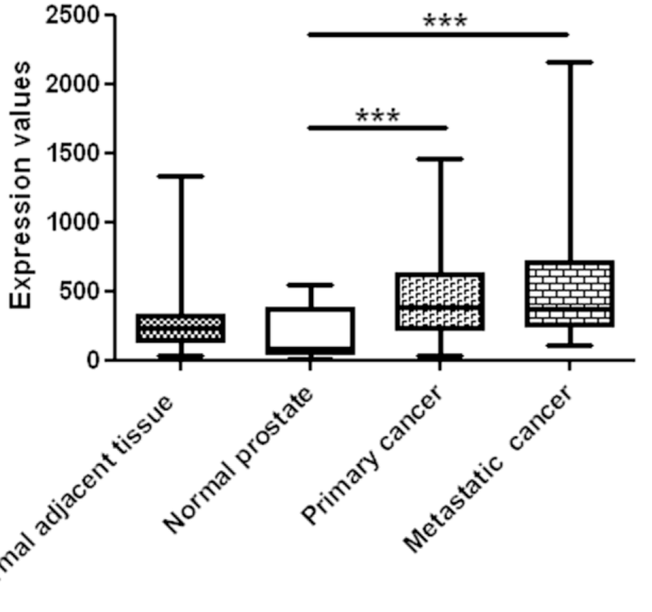

Supplementary Figure S1: Higher GPR160 expression in primary and metastatic prostate cancer samples presented by the gene expression omnibus (GEO) database (ID: 34871519). ****P* < 0.001 compared to normal prostate tissues.

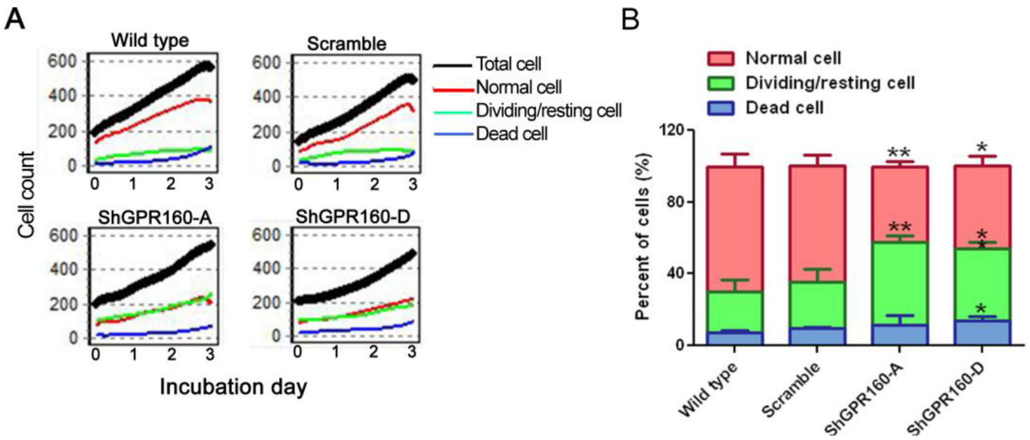

Supplementary Figure S2: Cell viability and morphological change affected by lentivrus-mediated knockdown of GPR160. The 22Rv1 cells were seeded at 2.5 × 10^4 cells per well onto 24-well plates 72 h after virus treatment and inserted into the Cell-IQ live cell imaging and analysis platform (CM Technologies location and country) with a long-time monitoring and a time lapse imaging for 72 h at 37°C, 5% CO_2. Two to three regions of cells with similar cell density from each well were imaged and cells with different morphology were counted (**A**). Cells with round morphology were marked as dividing or resting cells. (**B**) Distribution of normal, round and dead cell population at 48 h after cell seeding. Data were means ± SD of triplicate experiments. *P < 0.05, **P < 0.01 compared with scramble virus-infected cells.

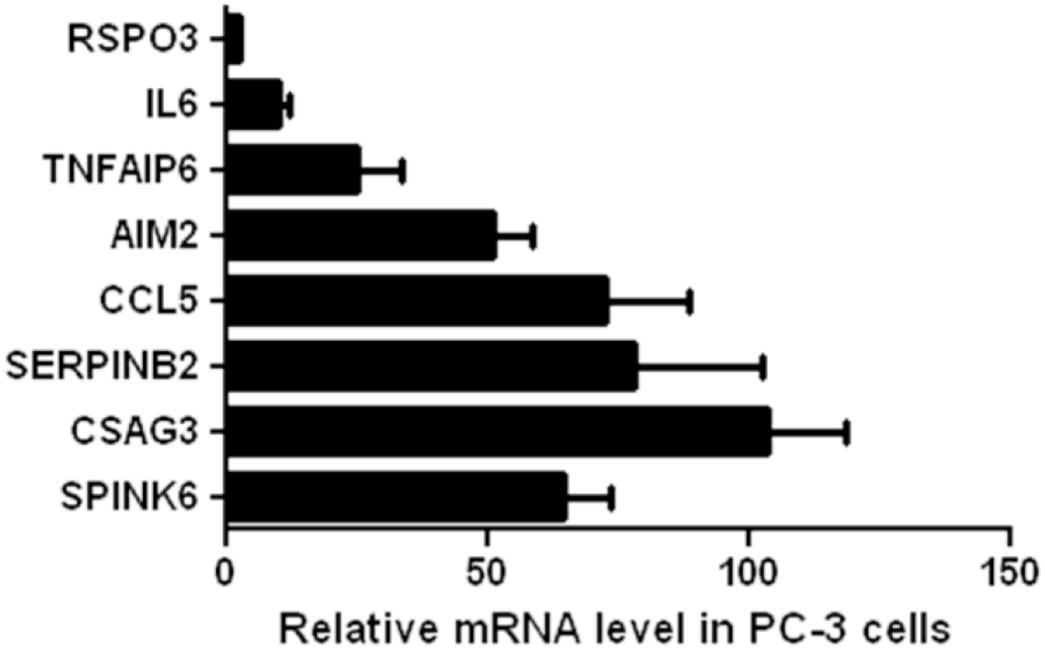

Supplementary Figure S3: Relative mRNA levels of differentially expressed genes between PC-3 cells treated with scramble and shRNAs targeting GPR160. Cells were collected 4 days after lentivirus transduction and subjected to quantitative RT-PCR analysis. Bars depict means ± s.e.m. of at least three independent experiments in triplicate.

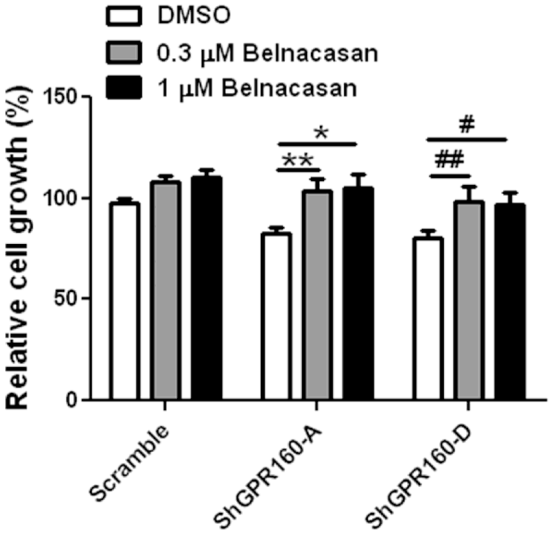

Supplementary Figure S4: Caspase 1 inhibitor rescues PC-3 cells from apoptosis induced by knockdown of GPR160. Cells were seeded at a density of 8000 cells per well and incubated overnight. Scramble or ShGPR160 lentiviruses were added to cells at an multiplicity of infection of 20 for 24 h followed by the incubation with indicated concentrations of Belnacasan or DMSO for another 4 days. Cell viability was evaluated with Cell Counting Kit-8. Bars depict means ± s.e.m. of three independent experiments in triplicate. $^{*,\#}P < 0.05$, $^{**,\#\#}P < 0.01$.

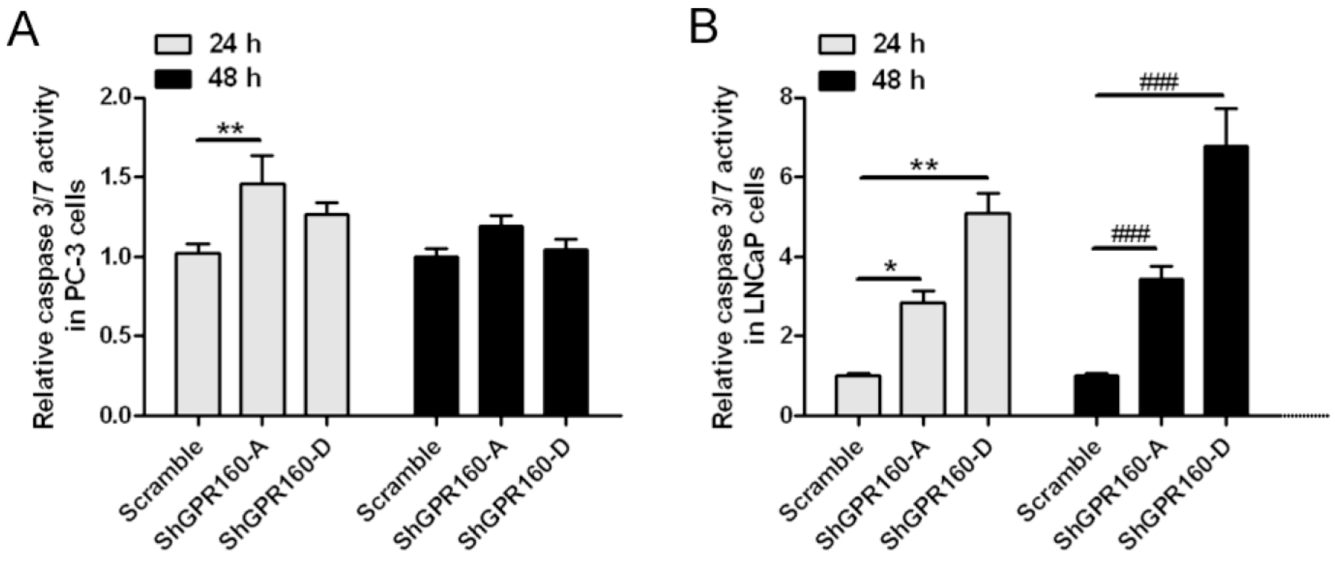

Supplementary Figure S5: Downregulation of GPR160 expression leads to increased caspase 3/7 activities in PC-3 and LNCaP cells. Cells were infected with scramble or ShGPR160 lentiviruses for 2 days, then seeded at a density of 500 wells per well in 384-well plates. After overnight incubation, the medium was replaced with RPMI1640 supplemented with 0.2% FBS and incubated for another 24 or 48 h. Caspase activity was subsequently measured with a Caspase-Glo 3/7 Assay System. Bars depict means ± s.e.m. of three independent experiments in triplicate. $^*P < 0.05$, $^{**}P < 0.01$, $^{,\#\#\#}P < 0.001$.

Supplementary Table S1: Primers used for quantitative RT- PCR

Gene	Forward primer (5′-3′)	Reverse primer (5′-3′)
**GPR160**	TGCAGTCCGGAGACGAACG	GTAAGCTCGGAGACATTTGCG
**SPINK6**	TGACTGTGGTGAGTTCCAGGA	CCACTTTTCACTATGGCCTTACA
**CSAG**	CCAACACCAAAGAGGTTCCCA	GGCTGTCCGAAGAGAGACTG
**SERPINB**	TGAGAAGTCTGCGAGCTT	TTGCCTTTGGTTTGAGTC
**CCL5**	CCAGCAGTCGTCTTTGTCAC	CTCTGGGTTGGCACACACTT
**AIM2**	CAGAAATGATGTCGCAAAGCAA	TCAGTACCATAACTGGCAAACAG
**TNFAIP6**	GGGCAGAGTTGGATACCCC	TGCGTGTGGGTTGTAGCAATA
**IL6**	ACTCACCTCTTCAGAACGAATTG	CCATCTTTGGAAGGTTCAGGTTG
**RSPO3**	TGTGCAACATGCTCAGATTACA	TGCTTCATGCCAATTCTTTCCA
**IL12A1**	ATGGCCCTGTGCCTTAGTAGT	AGCTTTGCATTCATGGTCTTGA
**JAK2**	ATCCACCCAACCATGTCTTCC	ATTCCATGCCGATAGGCTCTG
**CASP1**	GCCTGTTCCTGTGATGTGGAG	TGCCCACAGACATTCATACAGTTTC
**CDKN1A**	CTGCCCAAGCTCTACCTTCC	CAGGTCCACATGGTCTTCCT
**CCNB1**	CCTGGCTAAGAATGTAGTCATGGTAA	GCATGCTTCGATGTGGCATA
**CCNB2**	TTGGCTGGTACAAGTCCACTC	TGGGAACTGGTATAAGCATTGTC
**CDC25C**	GAACAGGCCAAGACTGAAGC	GCCCCTGGTTAGAATCTTCC
**ACTB**	GAGAAAATCTGGCACCACACC	ATACCCCTCGTAGATGGGCAC

Supplementary Table S2: List of top 20 up-regulated transcripts

Symbol	Gene name	Fold change	*P* value
**KRT17**	Keratin 17	+160.53	0.0036
**SPINK6**	Serine peptidase inhibitor, Kazal type 6	+122.03	0.0001
**CSAG3**	CSAG family, member 3	+112.41	< 0.0001
**KRT34**	Keratin 34	+104.45	0.0036
**SERPINB2**	Serpin peptidase inhibitor, clade B (ovalbumin), member 2	+59.50	0.0001
**CCL5**	Chemokine (C-C motif) ligand 5	+42.02	0.0099
**AIM2**	Absent in melanoma 2	+36.15	0.0024
**PDCD1LG2**	Programmed cell death 1 ligand 2	+35.54	0.0086
**TNFAIP6**	Tumor necrosis factor, α-induced protein 6	+28.12	0.0004
**CCL3L3**	Chemokine (C-C motif) ligand 3-like 3	+26.60	0.0264
**CXCL11**	Chemokine (C-X-C motif) ligand 11	+26.25	0.0038
**KRT6C**	Keratin 6C	+24.66	0.0298
**IL29**	Interleukin 29 (interferon, λ1)	+24.21	0.0128
**IL6**	Interleukin 6 (interferon,β2)	+21.75	< 0.0001
**RTP4**	Receptor (chemosensory) transporter protein 4	+19.76	0.0167
**LOC201651**	Arylacetamide deacetylase (esterase) pseudogene	+19.74	< 0.0001
**KRT16**	Keratin 16	+18.45	0.0215
**SLC15A3**	Solute carrier family 15, member 3	+18.11	0.0251
**RSPO3**	R-spondin 3 homolog (*Xenopus laevis*)	+17.72	0.0009

Supplementary Table S3: List of top 20 down-regulated transcripts

Symbol	Gene name	Fold change	*P* value
**MS4A7**	Membrane-spanning 4-domains, subfamily A, member 7	–19.12	0.0145
**HPGD**	Hydroxyprostaglandin dehydrogenase 15-(NAD)	–18.48	0.0003
**APOBEC3C**	Apolipoprotein B mRNA editing enzyme, catalytic polypeptide-like 3C	–16.47	< 0.0001
**NOX5**	NADPH oxidase, EF-hand calcium binding domain 5	–15.58	0.0009
**PALMD**	Palmdelphin	–13.34	0.0034
**COL2A1**	Collagen, type II, α1	–13.32	0.0056
**FABP4**	Fatty acid binding protein 4, adipocyte	–12.90	0.0163
**ARHGDIB**	Rho GDP dissociation inhibitor (GDI) β	–10.95	0.0497
**OLFML2A**	Olfactomedin-like 2A	–10.82	0.0148
**LFNG**	LFNG O-fucosylpeptide 3-beta-N-acetyl-Glucosaminyl transferase	–10.68	0.0076
**NNMT**	Nicotinamide N-methyltransferase	–10.05	0.0007
**RNF157**	Ring finger protein 157	–9.74	0.0004
**FAM131B**	Family with sequence similarity 131, member B	–9.64	0.0001
**TRA**	T cell receptor α locus	–9.45	0.0176
**SCNN1A**	Sodium channel, nonvoltage-gated 1 α	–8.90	0.0105
**VPS13D**	Vacuolar protein sorting 13 homolog D (*S. cerevisiae*)	–8.53	0.0165
**OGFRL1**	Opioid growth factor receptor-like 1	–8.36	0.0019
**CAPS**	Calcyphosine	–8.35	0.0123
**GNL1**	Guanine nucleotide binding protein-like 1	–8.27	0.0138
**CNGA1**	Cyclic nucleotide gated channel α1	–8.07	0.0049
